# Establishment and Characterization of a Novel Multidrug Resistant Human Ovarian Cancer Cell Line With Heterogenous MRP7 Overexpression

**DOI:** 10.3389/fonc.2021.731260

**Published:** 2021-09-24

**Authors:** Jing-Quan Wang, Zhuo-Xun Wu, Yuqi Yang, Jin-Sui Li, Dong-Hua Yang, Ying-Fang Fan, Zhe-Sheng Chen

**Affiliations:** ^1^ Department of Pharmaceutical Sciences, College of Pharmacy and Health Sciences, St. John’s University, Queens, NY, United States; ^2^ Department of Hepatobiliary Surgery, Zhujiang Hospital, Southern Medical University, Guangzhou, China

**Keywords:** ovarian cancer, multidrug resistance, ABCC10, MRP7, chemotherapy

## Abstract

Ovarian cancer is one of the leading female malignancies which accounts for the highest mortality rate among gynecologic cancers. Surgical cytoreduction followed by chemotherapy is the mainstay of treatment. However, patients with recurrent ovarian cancer are likely to exhibit resistance to chemotherapy due to reduced sensitivity to chemotherapeutic drugs. Adenosine triphosphate (ATP)-binding cassette (ABC) transporters have been extensively studied as multidrug resistance (MDR) mediators since they are responsible for the efflux of various anticancer drugs. Multidrug resistance protein 7 (MRP7, or ABCC10) was discovered in 2001 and revealed to transport chemotherapeutic drugs. Till now, only limited knowledge was obtained regarding its roles in ovarian cancer. In this study, we established an MRP7-overexpressing ovarian cancer cell line SKOV3/MRP7 *via* transfecting recombinant MRP7 plasmids. The SKOV3/MRP7 cell line was resistant to multiple anticancer drugs including paclitaxel, docetaxel, vincristine and vinorelbine with a maximum of 8-fold resistance. Biological function of MRP7 protein was further determined by efflux-accumulation assays. Additionally, MTT results showed that the drug resistance of the SKOV3/MRP7 cells was reversed by cepharanthine, a known inhibitor of MRP7. Moreover, we also found that the overexpression of MRP7 enhanced the migration and epithelial-mesenchymal transition (EMT) induction. In conclusion, we established an *in vitro* model of MDR in ovarian cancer and suggested MRP7 overexpression as the leading mechanism of chemoresistance in this cell line. Our results demonstrated the potential relationship between MRP7 and ovarian cancer MDR.

## Introduction

Ovarian cancer is one of the leading female malignancies in the United States. It accounts for approximately 2.5% of all cancers and 5% of cancer deaths among females. In 2018, there were approximately 22,000 new cases of diagnosed ovarian cancer and 14,000 ovarian cancer deaths according to the National Center for Health Statistics (1). Besides surgery, one of the most common treatments of ovarian cancer is chemotherapy (2). Over the past decades, the ovarian cancer chemotherapy regimens have evolved. Single-agent chemotherapy using melphalan was replaced by combinational treatment using the anthracycline doxorubicin and cisplatin (3). Then, in the early 1990s, paclitaxel was considered as the most effective agent in platinum-resistant ovarian cancer (3). Other taxanes such as docetaxel and *Vinca* alkaloid such as vinorelbine were also used in relapsed patients (2).

The ABC transporter superfamily is composed of 7 subfamilies (ABCA to ABCG). In the past two decades, extensive studies have been performed regarding the cancer multidrug resistance (MDR) mediated by ABC transporters. Major ABC transporters such as P-glycoprotein (P-gp, ABCB1), breast cancer resistance protein (BCRP, ABCG2) and multidrug resistance protein 1 (MRP1, ABCC1) have been proved to mediate the resistance to structurally distinct anticancer drugs (4–6). Multidrug resistance protein 7 (MRP7 or ABCC10) was first discovered in 2001 *via* expression tag mining. Further analysis revealed that MRP7 is a 173 kDa protein with three transmembrane domains (TMDs) and two nucleotide-binding domains (NBDs) (7). Additionally, the MRP7 gene was found broadly expressed in human tissues such as pancreas, kidneys, brain, lung, ovary, testis, prostate, colon, leukocytes and skin (8). Moreover, Chen et al. characterized the drug resistance profile of the MRP7. The results showed that MRP7 was responsible for mediating the drug resistance to taxanes, epothilones, alkaloids, anthracyclines and epipodophyllotoxins (9). The overlapping between MRP7-mediated MDR profile and common chemotherapeutic drugs for ovarian cancer inspired us to further explore the potential relationship between MRP7 overexpression and ovarian cancer MDR. Due to the close relationship between ABC transporters and the MDR in cancer, extensive studies have been carried out in modulating the efflux function of ABC transporters in order to overcome MDR and increase the chemotherapy efficacy (10). Proper MDR cancer cell models enable researchers to develop potent ABC transporter inhibitors as well as discovery new substrates. For example, recently a number of small molecules have been discovered as strong modulators of major ABC transporters such as ABCB1 (11–14), ABCG2 (15–17) and ABCC1 (18).

In this study, we successfully established an MRP7-overexpressing SKOV3/MRP7 cell line by transfecting recombinant pcDNA3.1/MRP7 plasmids. The MRP7 expression and subcellular localization was confirmed by Western blotting and immunofluorescence assay, indicating functional MRP7 was produced. We also found that MRP7 altered cell morphology and reduced cell-cell contact. Additionally, MRP7 overexpression promotes cell migration and up-regulating EMT marker N-cadherin. Moreover, the SKOV3/MRP7 cell line showed MDR to multiple chemotherapeutic drugs including paclitaxel, docetaxel, vincristine, vinorelbine, vinblastine but not to doxorubicin and cisplatin. The MDR could be reversed by co-treatment with a known MRP7 inhibitor cepharanthine. In conclusion, MRP7 could be closely relevant to the acquired drug resistance in ovarian cancer to several commonly used chemotherapeutic drugs.

## Methods

### Chemicals and Reagents

Chemotherapeutic drugs and reagents used in this study were purchased from Sigma Chemical Co if otherwise stated (St. Louis, MO) including paclitaxel, docetaxel, vincristine, vinorelbine, vinblastine, doxorubicin, formaldehyde, Triton X-100, 3-(4, 5-dimethylthiazol-yl)-2, 5-diphenyltetrazolium bromide (MTT) and anti-MRP7 antibody (HPA041607) produced in rabbit. Cisplatin and geneticin (G418) were purchased from Enzo Life Sciences (Farmingdale, NY). Recombinant MRP7 plasmid was prepared as previously described (19). The [^3^H]-paclitaxel was purchased from Moravek Biochemicals, Inc (Brea, CA). Fetal bovine serum (FBS), RMPI1640 medium, and 0.25% trypsin-EDTA were ordered from Corning Inc. (New York, NY). Phosphate buffer saline (PBS), dimethyl sulphoxide (DMSO), the Alexa Fluor 488-labeled secondary antibody (anti-mouse) and 4,6-diamidino-2-phenylindole (DAPI) were ordered from Thermo Fisher Scientific Inc. (Rockford, IL).

### Cell Lines and Cell Culture

The human ovary adenocarcinoma cell line SKOV3 was purchased from ATCC (Manassas, VA). The HEK293 and HEK293/MRP7 cell line were established and maintained as previously described (20). Both transfected cells were selected and cultured in RMPI1640 supplemented with 10% FBS and 2 mg/mL G418 in a 5% CO_2_ incubator at 37°C. Cell morphology was observed using optical microscope.

### Recombinant MRP7 Plasmid Transfection

The recombinant expression vector of MRP7 was established based on pcDNA3.1 plasmid as previously described (19). Transfection of the empty or recombinant vector into SKOV3 was performed using Fugene6 transfection agent (Promega, Madison, WI) following the manufacturer’s instructions. In brief, SKOV3 cells were seeded in 6-well plates with 100,000 to 200,000 cells per well with RMPI1640 with 10% FBS. Then, 100 μl mixture of plasmid DNA and Fugene6 reagent (1:3 DNA : Fugene6 ratio) was prepared and incubated at room temperature for 30 min. Then, the mixture was added into cell culture medium and incubated with cells for 2 days. When incubation ends, cell culture medium with transfection reagent was removed and transfected cells were rinsed with PBS. Selection medium (RMPI1640, 10% FBS and 2 mg/ml G418) was added and incubated with cells for at least 14 days. Survived cells that formed single colonies were collected and cultured separately. The expression of MRP7 was further verified by Western blotting and immunofluorescence assay. In this paper, we use “SKOV3” to represent empty-vector-transfected “SKOV3/pcDNA3.1” unless otherwise stated.

### Population Doubling Time (PDT) Assessment

The PDT was used to examine the cell growth rate of SKOV3 and SKOV3/MRP7. Cells were plated evenly into a T25 flask at the density of 1,000,000 cells/flask and cultured at 37 °C. Cells were harvested and counted each day for a period of 6 days. Trypan blue was used to determine the average number of living cells on each day. The cell growth curve was plotted as log(N) *versus* time, where N is the average live cell count. The linear portion of the cell growth curve, which represents exponential growth, was subjected to PDT calculation using the following equation: PDT = T×log (2)/log (N1/N0). In this equation, T is the culture time, N1 is the cell number at the end of the culture period, N0 is the cell number at the beginning of the culture period.

### Cell Viability Assay

The cell viability was measured by a modified MTT assay as previously described with slight modifications (21). In brief, cells were seeded at a density of 6000-8000 cells/well in 96-well plates 24 h prior to adding drugs. Then, cells were treated with different concentration of chemotherapeutic drugs for 72 h. For reversal study, MRP7 inhibitor cepharanthine was added to the 96-well plates 2 h before adding a chemotherapeutic drug. At the end of the treatment, cell viability was determined by an MTT assay. Resistance fold was determined as fold relative to the parental control group. The half maximal effect concentrations (EC_50_) were calculated using the regression algorithm provided in GraphPad Prism 8.

### Wound Healing Assay

SKOV3 and SKOV3/MRP7 cells were seeded into 24-well plates at the density of 500,000 cells/well and incubated overnight. At the following day, the cells were washed with PBS and then a 200 μL pipet tip was used to create a scratch by scraping the cell monolayer in a straight line. The cells were then incubated in RPMI1640 medium with 5% FBS, and the images were captured at 0, 8, 12 and 24 h.

### Western Blotting

Western blotting was performed as previously described with slight modifications (22). The membrane and cytoplasm proteins were separated using Thermo Scientific Mem-PER Plus Membrane Protein Extraction Kit (Thermo Fisher Scientific, Waltham, MA) and followed the manufacturer’s instruction. The protein mixture was separated using SDS-polyacrylamide gel electrophoresis (SDS-PAGE) and electro-transferred to a polyvinylidene difluoride (PVDF) membrane. Then, the membrane was blocked by 5% non-fat milk for 2 h at room temperature. The primary antibodies (1:1000 dilution) of MRP7 (HPA041607), N-Cadherin (Catalog#14215, Cell Signaling, Danvers, MA), E-Cadherin (Catalog# 3195S, Cell Signaling, Danvers, MA), membrane protein loading control Caveolin-1 (Catalog# MA3-600, Thermo Fisher Scientific Inc., Waltham, MA) and GAPDH were incubated with the blocked membrane at 4°C overnight. After rinsed with TBST for 3 times (15 min each), the membrane was incubated in the secondary HRP-linked antibody (1:1000 dilution) for 2 h at room temperature. Blotted protein bands were visualized using an enhanced ECL kit (Thermo Fisher Scientific Inc., Waltham, MA).

### Immunofluorescence Assay

In brief, SKOV3 and SKOV3/MRP7 cells were seeded in 24-well plates at a density of 50,000 cells/well and cultured overnight. Immunofluorescence assay was performed as previously described with slight modifications (15). In brief, cells were processed with 4% formaldehyde (37°C for 15 min) and 0.1% Triton X-100 (37°C for 15 min). Cells were rinsed with cold PBS before adding the next reagent. Next, cells were incubated with primary anti-MRP7 (1:200) at 4°C overnight followed by incubation with Alexa Fluor 488 conjugated secondary antibody (1:1000) for 2 h at 37°C. DAPI was used to visualize the nuclei. Images were taken using a fluorescence microscope.

### Paclitaxel Accumulation and Efflux Assay

Paclitaxel accumulation and efflux assay were performed as previously described with slight modifications (23). In brief, cells were seeded with a density of 10,000 cells/well into 24-well plates and cultured overnight. Then, cells were incubated in medium containing 10 nM [^3^H]-paclitaxel at 37°C for 2 h. After incubation, cells were rinsed with cold PBS and incubated in [^3^H]-free medium for 0, 0.5, 1 and 2 h. At each time point, cells were detached and transferred into 5 mL scintillation fluid. The radioactivity was measured using a Tri-Carb liquid scintillation counter (Packard Instrument Inc., Chicago, IL). The [^3^H]-paclitaxel level at 0 h was used to represent the intracellular accumulation within 2 h incubation. The change in [^3^H]-paclitaxel levels at following time points were used to measure the efflux activity.

### Statistical Analysis

Comparison of differences among groups were performed using one-way ANOVA. The statistical criteria p < 0.05 was considered as statistically significant. All data were represented as mean ± SD from at least three independent experiments.

## Results

### Verification of the MRP7 Expression and Paclitaxel Resistance in G418-Selected SKOV3/MRP7 Cells

**Figure 1:**
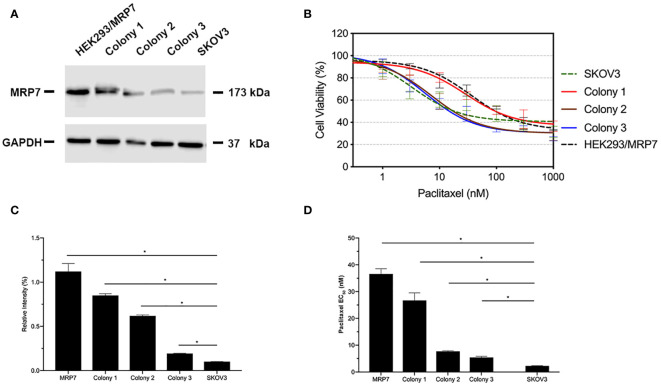
MRP7 expression and paclitaxel cytotoxicity in SKOV3/MRP7 cells. HEK293/MRP7 and SKOV3 were used as positive and negative controls respectively. **(A)** Western blotting results showing MRP7 expression of selected colonies and controls. **(B)** Cytotoxicity of paclitaxel in selected colonies and controls. **(C)** Semi-quantitative analysis of MRP7 protein expression showed in **(A)**. **(D)** Paclitaxel EC_50_ in selected colonies and controls. In **(C)** and **(D)**. “MRP7” stands for “SKOV3/MRP7”. ^*^p < 0.05 *versus* the negative control group.

The transfected cells were cultured in selection medium containing 2 mg/ml G418 for 2 weeks. Survived cells formed single colonies. Three colonies were picked, then cultured in G418-free medium for an extra week. Western blotting was performed when cells were about 80% confluent. Results were shown in **Figure 1**. According to the results in **Figures 1A, C**, colony 1 exhibited the highest MRP7 expression. Moreover, it is worth noting that endogenous MRP7 expression was also detected in parental SKOV3 cells, even though the expression level was low. Furthermore, we examined the cytotoxicity of paclitaxel, a known substrate of MRP7, to see if the selected cell lines become resistant to paclitaxel. Based on the results in **Figures 1B, D**, only colony 1 showed significantly increased paclitaxel EC_50_, indicating the acquired resistance from MRP7 overexpression. To further analyze the drug resistance profile of the new SKOV3/MRP7 cell line, we chose colony 1 for following steps. To summarize, we successfully transfected *MRP7* gene into SKOV3 cells and the transfected cell line showed MRP7-mediated paclitaxel resistance.

### MRP7 Overexpression and the Growth, Morphology and Migration of SKOV3 Cells

**Figure 2:**
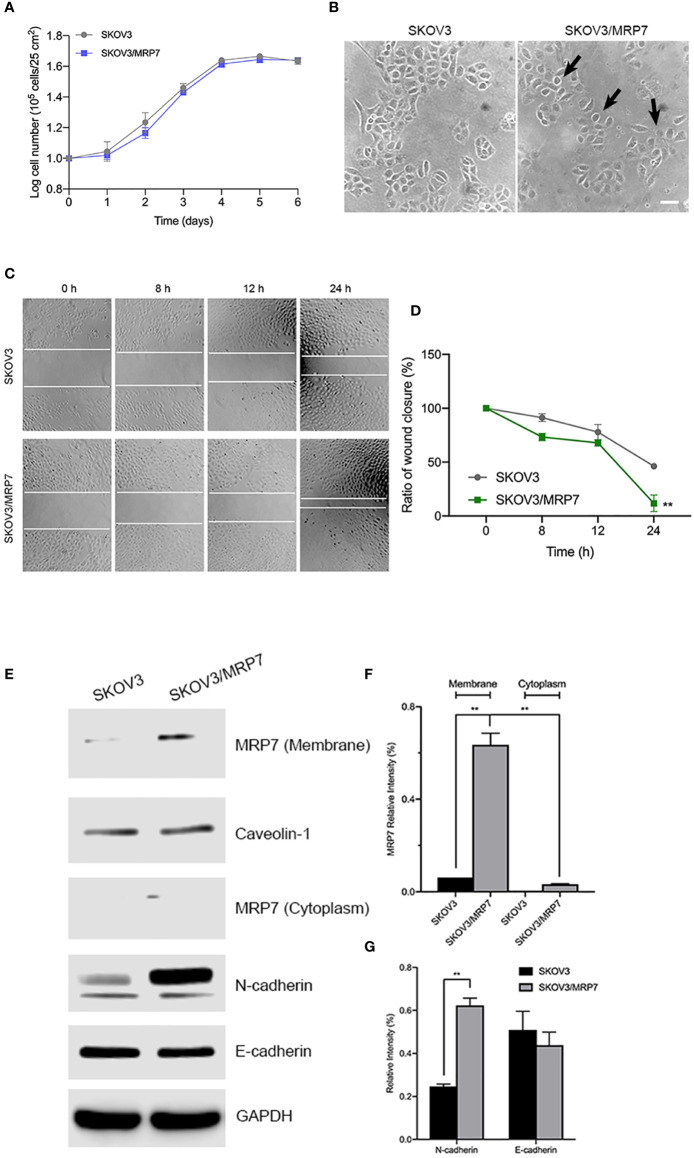
Morphology, migration and EMT marker expression of SKOV3 and SKOV3/MRP7 cells. **(A)** Growth curve of SKOV3 and SKOV3/MRP7 cells. **(B)** Cell morphology of SKOV3 and SKOV3/MRP7 cells under optical microscope. Scale bar = 100 μm. Black arrows indicating reduced cell-cell contact. **(C)** Wound healing assay of SKOV3 and SKOV3/MRP7 cells. White solid line indicates the edges. **(D)** Semi-quantification of wound healing assay. Grey and green solid line represent the ratio of wound closure of SKOV3 and SKOV3/MRP7 cells, respectively. **(E)** Western blotting of membrane/cytoplasm protein and EMT markers. **(F)** Semi-quantification of membrane/cytoplasm protein. **(G)** Semi-quantification of EMT marker expression. ^**^p < 0.01 *versus* the negative control group. Data are expressed as mean ± SD derived from three independent experiments.

To evaluate the effect of MRP7 overexpression on various biological characteristics of SKOV3 cells, we first measured the growth of MRP7-expressing SKOV3 cells. The results in **Figure 2A** showed that MRP7 did not alter the cell growth. The PDT of SKOV3 was 68.33 ± 1.73 h while SKOV3/MRP7 was 68.19 ± 1.53 h. Additionally in **Figure 2B**, we found that MRP7-overexpressing SKOV3 cells displayed reduced cell-cell contact and exhibited rounder shaped, while the control SKOV3 cells had a more cobblestone-like cell morphology with tighter clusters. Previous results had shown that overexpression of ABC transporters altered the cell migration and EMT induction (24). As a result, we tested whether MRP7 had similar effects. The wound healing assay (**Figures 2C, D**) showed that MRP7 significantly stimulated cell migration in SKOV3/MRP7 cells. Moreover, Western blotting of EMT markers expression demonstrated that MRP7-induced cell migration is potentially relevant to increased N-cadherin in SKOV3/MRP7 cells (**Figures 2E, G**). The results indicated that MRP7 may alter the morphology and migration of ovarian cancer cells without altering the cell growth rate.

### Subcellular Localization of MRP7

**Figure 3:**
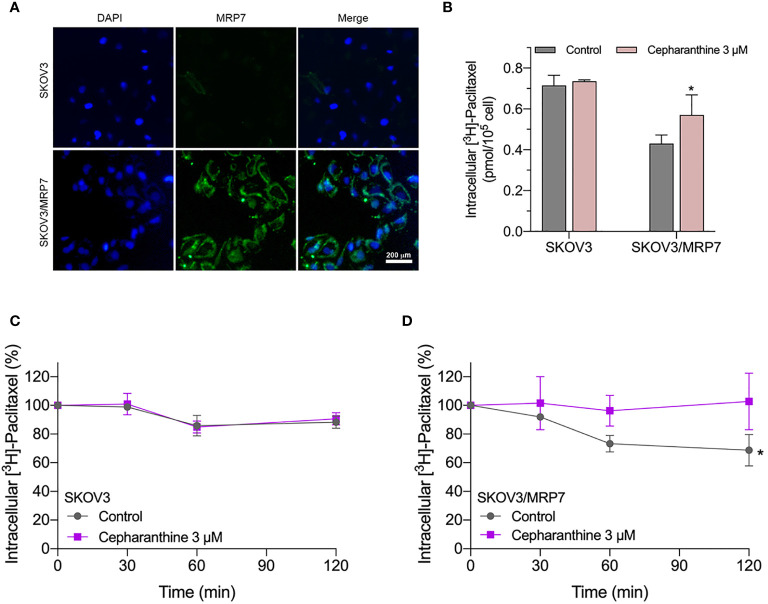
Immunofluorescence assay and accumulation-efflux assays of colony 1. **(A)** Subcellular localization of MRP7 in SKOV3 and SKOV3/MRP7 cells. **(B)** The intracellular accumulation of [^3^H]-paclitaxel in SKOV3 and SKOV3/MRP7 cells. **(C)** The efflux of [^3^H]-paclitaxel in SKOV3 cells. **(D)** The efflux of [^3^H]-paclitaxel in SKOV3/MRP7 cells. Data in **(B–D)** are expressed as mean ± SD derived from three independent experiments. ^*^p < 0.05 *versus* the control groups.

Similar to other ABC transporters, MRP7 requires membrane localization for efflux function. Therefore, we examined the subcellular localization of SKOV3/MRP7 cells *via* immunofluorescence assay. According to **Figure 3A**, in parental SKOV3 cells, no detectable green fluorescence was observed under the same parameters, which was consistent with the low expression level of endogenous MRP7 in SKOV3 cells. Strong green fluorescence was observed on the membrane of SKOV3/MRP7 cells, suggesting that the overexpressed MRP7 is localized on the cell membrane. To further confirm the subcellular localization of MRP7, we performed Western blotting using separated cytoplasm protein and membrane protein. As shown in **Figures 2E** (row 1 - 4) and 2F, MRP7 was mainly found in membrane but not in cytoplasm protein. Overall, our results showed that the MRP7 protein majorly located on cytoplasm membrane.

### Intracellular Accumulation of [^3^H]-Paclitaxel in SKOV3 and SKOV3/MRP7

We measured the intracellular accumulation of [^3^H]-paclitaxel in SKOV3 and SKOV3/MRP7 after incubation in [^3^H]-paclitaxel-containing culture medium for 2 h. The results in **Figure 3B** showed significantly different levels of intracellular accumulation of paclitaxel in SKOV3 and SKOV3/MRP7 cells. Specifically, SKOV3/MRP7 showed lower intracellular accumulation of paclitaxel than SKOV3. Moreover, in SKOV3/MRP7, the reduced paclitaxel accumulation was significantly reversed by MRP7 inhibitor cepharanthine. Above findings are consistent with previous MTT results that SKOV3/MRP7 was less sensitive to paclitaxel due to the overexpression of MRP7 protein.

### Efflux of [^3^H]-Paclitaxel in SKOV3 and SKOV3/MRP7

In the previous section, we found SKOV3/MRP7 cells showed lower paclitaxel intracellular accumulation, which could be significantly antagonized by MRP7 inhibitor cepharanthine. We then determined the efflux of paclitaxel by measuring the decreased intracellular amount at 0, 0.5, 1 and 2 h. Results in **Figures 3C, D** showed that by the end of the 2-h incubation, the intracellular concentration of paclitaxel was decreased by approximately 15% in SKOV3 cells. While in SKOV3/MRP7, intracellular paclitaxel concentration was decreased by approximately 40%, indicating strong efflux of paclitaxel mediated by MRP7. The efflux function of MRP7 was inhibited by cepharanthine effectively as paclitaxel accumulation similar to the parental cell line was restored. Overall, the accumulation/efflux assay results together showed that the MRP7 in transfected SKOV3 cells exhibited active drug efflux function which could be antagonized by cepharanthine, a known MRP7 inhibitor.

### The Drug Resistance Profile of MRP7-Overexpressing Ovarian Cancer Cell Line

**Figure 4:**
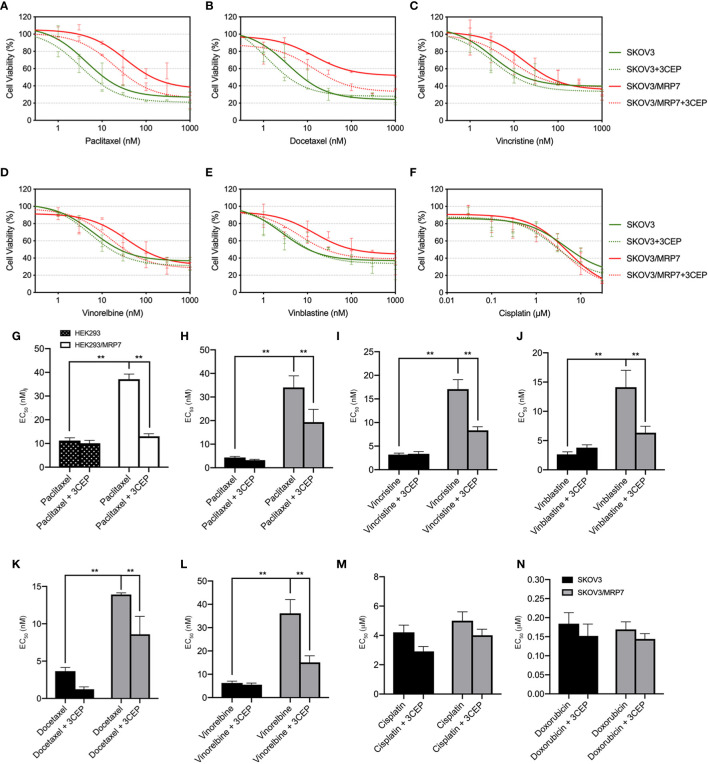
Reversal of MRP7-mediated drug resistance using cepharanthine in SKOV3 and SKOV3/MRP7 cells. Chemotherapeutic drug *versus* cell viability was plotted. **(A)** paclitaxel; **(B)** vinorelbine; **(C)** docetaxel; **(D)** vinblastine; **(E)** vincristine; **(F)** cisplatin. Green solid lines represent SKOV3, green dashed lines represent SKOV3 + 3 μM cepharanthine, red solid lines represent SKOV3/MRP7, red dashed lines represent SKOV3/MRP7 + 3 μM cepharanthine. **(G)** EC_50_ values of paclitaxel in HEK293 and HEK293/MRP7 with or without 3 μM cepharanthine. Black bar with white spots: HEK293; white bars: HEK293/MRP7. **(H–N)** EC_50_ values of chemotherapeutic drugs in SKOV3 and SKOV3/MRP7 with or without 3 μM cepharanthine: **(G)** paclitaxel; **(H)** vincristine; **(I)** vinblastine; **(J)** docetaxel; **(K)** vinorelbine; **(L)** cisplatin; **(M)** doxorubicin. Black bars: SKOV3; grey bars: SKOV3/MRP7. CEP: cepharanthine. ^**^p < 0.01 *versus* the SKOV3 or CEP groups. Data are expressed as mean ± SD derived from three independent experiments.

In previous sections, we have confirmed the successful establishment of an MRP7-overexpressing SKOV3 cell line. MTT assay and accumulation-efflux assays have confirmed the expression and biological function of the MRP7 protein. To further understand the drug resistance profile of the MRP7-overexpression ovarian cancer cell line, we performed MTT assay to test the sensitivity to commonly used chemotherapeutic drugs. Results were displayed in **Figures 4H–N**. Specifically, SKOV3/MRP7 showed significant resistance to paclitaxel (7.91-fold resistance), docetaxel (3.82-fold resistance), vincristine (5.33-fold resistance), vinorelbine (5.76-fold resistance) and vinblastine (5.34-fold resistance). Moreover, SKOV3/MRP7 showed no significant resistance to ABCB1/ABCG2 substrate doxorubicin. Also, SKOV3/MRP7 was not resistant to non-ABC-transporter-substrate cisplatin. Furthermore, **Figure 4G** showed the paclitaxel resistance profile of HEK293 and HEK293/MRP7. Comparing with SKOV3 and SKOV3/MRP7 (**Figure 4H**), it is clear that SKOV3/MRP7 exhibited similar paclitaxel resistance fold as HEK293/MRP7. Previous studies also reported consistent paclitaxel resistance of HEK293/MRP7 (7, 20, 25). As a result, our results further demonstrated the biological function of MRP7 in SKOV3/MRP7 cells.

### MRP7 Inhibitor Cepharanthine Antagonizes the Drug Resistance Phenotype of SKOV3/MRP7 Cells

To further confirm that the overexpression of MRP7 is the major contributing factor of drug resistance, we performed MTT assay with co-treatment of chemotherapeutic drugs and MRP7 inhibitor cepharanthine. As shown in **Figures 4A–F**, cell viability curves of SKOV3/MRP7 showed left-shifting when adding cepharanthine, indicating resistance to known MRP7-substrate drugs were reversed in SKOV3/MRP7 by cepharanthine. Specifically, significant changes were observed in the EC_50_ of paclitaxel (from 34.05 nM to 19.34 nM), docetaxel (13.92 nM to 8.60 nM), vincristine (17.08 nM to 8.34 nM), vinblastine (14.12 nM to 6.34 nM) and vinorelbine (36.16 nM to 15.12 nM). Graphical representation was provided in **Figure 4H–N**. The cell viability of parental SKOV3 cells were not significantly affected by cepharanthine. Cisplatin was used as a negative control since it’s not a substrate of major ABC transporters. Cell viability of both SKOV3 and SKOV3/MRP7 cells were not altered. The above results further demonstrated that the MDR phenotype was mainly caused by MRP7 overexpression in SKOV3/MRP7 cells.

## Discussion

Over the past decade, progression in the treatment of ovarian cancer has been exponentially gained (3). While new opportunities come with new challenges such as selecting the optimal treatment strategy and prolong patients’ life as much as possible. Currently, various strategies are available for the treatment of ovarian cancer such as immune-therapy and chemotherapy. Among all potential therapeutic choices, chemotherapy is still the major way to control the cancer progression (2). However, cancer MDR has been one of the major obstacles in successful chemotherapy, which was almost inevitable (26). MDR decreases the anticancer efficacy of chemotherapeutic agents (27). ABC transporters are membrane proteins responsible for the cross-membrane transportation of wide spectrum of endo- and xenobiotics (28). Thus cancer MDR is often associated with overexpression of ABC transporters such as ABCB1, ABCG2 and ABCC1, which have been proved to mediate the efflux of structurally distinct chemotherapeutic agents (28). As a result, understanding the relationship between ABC transporter and MDR in cancer is critical for improving the efficacy of chemotherapy. Till now, tremendous efforts have been made to explore the mechanism of MDR, mostly by establishing ABC transporter-overexpressing cancer cell lines (9, 29). By comparing parental and resistant cancer cell lines using molecular biology and cellular methods, researchers have identified numerous ABC transporter substrates (21, 30–34) as well as inhibitors (16, 35–40). Computational strategies were also developed to boost the modulator discovery process especially multitarget modulators (41, 42). Thus, establishing new cancer cell lines that overexpresses ABC transporters, especially those with limited knowledge like MRP7, will greatly enhance our understanding of MDR in cancer.

MRP7 was a relatively new ABC transporter first discovered in 2001 (19). Later, it has been proved to be associated with acquired MDR and the prognosis of colorectal cancer and lung cancer (43–45). Moreover, strong correlation between the mRNA level of MRP7 and forkhead box transcription factor protein (FOXM) indicated that MRP7 played critical roles in 5-fluorouracil resistance in colorectal cancer patients induced by FOXM (46). Furthermore, MRP7 has been shown to participate in vinorelbine resistance in lung cancer cells (7). These previous studies further support the importance in understanding how MRP7 is involved in MDR in various types of cancers.

Several common chemotherapeutic drugs for ovarian cancer such as taxanes and *Vinca* alkaloid were known to be MRP7 substrates (9). As a result, MRP7-overexpressing cells would confer resistance to these drugs. Due to the lack of proper cancer cell models, the relationship between MRP7 expression and ovarian cancer MDR remains unclear (47). In the current study, we successfully established an MRP7-overexpressing ovarian cancer cell line by transfecting recombinant pcDNA3.1/MRP7 plasmid. Using this ovarian cancer cell model, we showed that MRP7-overexpressing cells exhibited significant resistance to chemotherapeutic drugs such as taxanes and *Vinca* alkaloids. Additionally, the drug resistance could be reversed by MRP7-inhibitor cepharanthine (48), though only partially, indicating MRP7 as a key MDR factor in transfected SKOV3 cells. Hopper-Borge et al. also reported similar drug resistance phenotype in HEK/MRP7 cells, which further supported our conclusions (9). Since parental SKOV3 cells exhibited endogenous MRP7 expression, the up-regulation of MRP7 expression could be a risk factor of acquiring MDR in ovarian cancer. Previous studies have shown that overexpression of ABC transporters (such as ABCF1) not only altered the chemoresistance profile of cancer cells, but also affected the biological characteristics, such as morphology and migration (24), which are critical factors in cancer development. In our study, we also found that MRP7 altered the cell morphology and promoted cell migration *via* upregulating the EMT marker N-cadherin. Although functionally active ABC transporters have generally been observed in both the cell membrane and intracellular vesicles, MRP7 in particular has been demonstrated earlier to exert its main effect at the cell membrane (49). The immunofluorescence assay and membrane/cytoplasm protein immunoblotting showed that the MRP7 protein mainly localized on cell membrane. Since EMT is critical in cancer development which enables acquired aggressiveness by inducing cell motility, invasiveness as well as chemoresistance. Our findings suggest that MRP7 was not only involved in chemoresistance as a drug efflux transporter, but also facilitated the migratory potential of ovarian cancer. Further studies will be required to explore the relationship between MRP7 overexpression and EMT process.

In summary, our work showed that overexpression of MRP7 may be an important mechanism in acquired resistance to paclitaxel and other chemotherapeutic agents in ovarian cancer. Also, MRP7 overexpression could affect cell migration and EMT. However, the MDR mechanism in ovarian cancer is complex and further study is required to explore the molecular mechanism of MRP7-mediated MDR, our finding emphasized the importance of MRP7 in the development of ovarian cancer drug resistance. Additionally, establishment of this MRP7-overexpressing ovarian cancer cell line may facilitate the discovery of novel modulators to overcome acquired MDR in ovarian cancer and improve the therapeutic efficacy in cancer patients.

## Data Availability Statement

The raw data supporting the conclusions of this article will be made available by the authors, without undue reservation.

## Author Contributions

Conceptualization, J-QW and Z-SC. Methodology, J-QW, Z-XW, YY, and J-SL. Writing – original draft preparation, J-QW. Writing – review & editing, Z-XW, D-HY, and Z-SC. Supervision, YFF and Z-SC. All authors contributed to the article and approved the submitted version.

## Funding

This study is supported by the Key-Area Research and Development Program of Guangdong Province, China (2020B010165004).

## Conflict of Interest

The authors declare that the research was conducted in the absence of any commercial or financial relationships that could be construed as a potential conflict of interest.

## Publisher’s Note

All claims expressed in this article are solely those of the authors and do not necessarily represent those of their affiliated organizations, or those of the publisher, the editors and the reviewers. Any product that may be evaluated in this article, or claim that may be made by its manufacturer, is not guaranteed or endorsed by the publisher.
